# Unmasking unexpected health care inequalities in China using urban big data: Service-rich and service-poor communities

**DOI:** 10.1371/journal.pone.0263577

**Published:** 2022-02-10

**Authors:** Linzi Zheng, Lu Zhang, Ke Chen, Qingsong He

**Affiliations:** 1 College of Public Administration, Huazhong University of Science and Technology, Wuhan, China; 2 School of Public Administration, Central China Normal University, Wuhan, China; 3 School of Civil and Hydraulic Engineering, Huazhong University of Science & Technology, Wuhan, China; Xiamen University, CHINA

## Abstract

Geographic accessibility plays a key role in health care inequality but remains insufficiently investigated in China, primarily due to the lack of accurate, broad-coverage data on supply and demand. In this paper, we employ an innovative approach to local supply-and-demand conditions to (1) reveal the status quo of the distribution of health care provision and (2) examine whether individual households from communities with different housing prices can acquire equal and adequate quality health care services within and across 361 cities in China. Our findings support previous conclusions that quality hospitals are concentrated in cities with high administrative rankings and developmental levels. However, after accounting for the population size an “accessible” hospital serves, we discern “pro-poor” inequality in accessibility to care (denoted as GAPSD) and that GAPSD decreases along with increases in administrative rankings of cities and in community ratings. This paper is significant for both research and policy-making. Our approach successfully reveals an “unexpected” pattern of health care inequality that has not been reported before, and our findings provide a nationwide, detailed benchmark that facilitates the assessment of health and urban policies, as well as associated policy-making.

## Introduction

The problem of health care inequalities in China became noticeable after the turn of the new millennium, i.e., 20 years after the country established economic liberalization in the 1980s. The health care system during that period was alleged to be market-based without a ‘safety net’, and it created much inequality [1]. Public discontent with limited access to care and soaring expenditures (called *Kan-bing-nan* and *Kan-bing-gui* by the Chinese press) grew and finally prompted a major health care reform in 2009 [2]. The new health care system emanating from the complex 2009 reform was ambitiously pitted against the previous one; it put great emphasis on redefining the role of the state through ‘flagship’ moves such as promoting primary health care, expanding the coverage of social health insurance, working off price markups of drug sales, innovating public hospitals and promoting diversified channels of health care provision [3, 4]. The ultimate goal was to align domestic health care with broader trends of universal health coverage (UHC) worldwide [5].

However, the efficacy of China’s new system in alleviating health care inequalities has been continuously questioned, particularly after the launch of a conspicuous policy in 2012. The government announced the goal of a fifth private hospital share by 2015, implicitly inviting competition from the private sector for health care provisions. Some researchers described this policy as an agenda spurring the new system into a ‘government-market pendulum’ guiding service delivery, adding to challenges facing China in promoting UHC [4]. Such an anfractuous situation motivated both a fierce ideological debate and empirical studies on China’s health care inequalities in varying terms and at different levels of granularity [2, 6, 7].

In copious research on health care inequalities across the globe, accessibility is a frequently cited concept [8–10]. Patterns of unequal accessibility have been studied from both the demand side (e.g., use and affordability of health services [11, 12]) and the supply side (e.g., differences in proximity to care, availability, and distribution of resources [13–15]). Among these supply-side differences, spatial accessibility is a classic measure for assessing health care inequality [9, 11, 16]. This is not only because “time is life” for acute incidents such as cardiovascular disease and road trauma, but many health care systems offer limited services, meaning that the spatial configuration of health care facilities effectively creates ‘constituencies’ for each [17]. Thus, for populations defined by places of residence, proximities to care under different supply-demand conditions play an important role in segregating their use of health services, thereby producing health care inequalities [10].

In China, spatial analyses of health care inequalities have attracted intensive attention [5, 18–24]. These studies, however, are based on data sourced from censuses or yearbooks; they are best at a provincial scale and with granularity finest at a street level, or on a national scale but lacking information on interarea variations. Thus, the available findings do not reveal much about health care inequalities in a spatially specific, context-extensive manner. Moreover, studies using data from censuses or yearbooks tend to lose sight of the importance of accuracy in the computation of supply and demand. A recent study by [24] pointed out that the official data on hospitals, which are available in the National Health and Family Planning Commission (NHFPC) database, are incomplete. Hospitals, such as those affiliated with major ministries and the Ministry of Public Security, are not included due to limited statistical coverage as a result of fragmented arrangements of “*kuai*” (i.e., jurisdiction boundaries between Central and local governments) and “*tiao*” (i.e., jurisdiction boundaries between local governments). Additionally, the data on potential demand in existing studies—population—is generally from the registered permanent population that may well be inconsistent with the real-time population since such data are ideally updated every six months, not to mention that the latest published census is version 2010. Hence, existing studies have failed to complete the knowledge about the status quo of China’s health care inequalities more than 10 years after the 2009 reform began. Chief among the lacuna is a national panorama of locally-specific, accurate depiction of health care inequalities under varying location-dependent supply-demand conditions.

In this paper, the specified unknown is trenchantly addressed by the merits of nationwide, community-based biggish data and geospatial approaches to health care accessibility for different populations across urban China. We have employed a fairly straightforward measurement of accessibility—spatial proximity to care—to probe health care inequality. The novelty is that we map countrywide evidence of whether accessibility to quality health care is equal based on both the wealth status and size of the target audience, i.e., normally residents in one or more communities. Our approach applies well to China’s situation, not only because the health care system here is largely community-based but also because hospitals of high quality and in close proximity tend to “subjugate” patients [23]. We focus on quality services provided by either public or private hospitals, while relatively inferior hospitals and clinical services, as helpful as they are, are not included in our analysis. We also do not consider the varying needs of different groups and their actual use of services nor do we extrapolate policy recipes from health outcomes. Rather, the primary contribution of this research is to offer a spatial benchmark of health care inequalities in a much broader context and with finer details than before, against which the impact of future policies can be assessed in a more specific way. Bringing such a benchmark into light is far from inconsequential. Urban areas with an urgent need to address inequalities can be more easily identified, facilitating not only scholarly research but also the contextualization of prospective urban health policies that may result in national and local improvements for years to come.

## Materials and methods

### Study area

Urban areas in China are defined differently by professionals such as urbanists, geographers, and sociologists. Here, we define urban areas following China’s administrative divisions and probably render the widest range of sample cities (a total of 361), including 4 direct-controlled municipalities with provincial administrative level, 28 provincial capitals (prefecture-level cities except Guangzhou, Wuhan, Harbin, Shenyang, Chengdu, Xi’an, Changchun, Jinan, Nanjing and Hangzhou that are subprovincial-level cities, i.e., cities that are half a level above prefecture-level cities), the remaining 5 subprovincial-level cities (Shenzhen, Dalian, Qingdao, Ningbo and Xiamen that enjoy economic autonomies similar to provincial cities), 306 non-provincial capital cities at the prefecture level (including 280 cities and 26 prefectures, leagues and autonomous prefectures) and 23 county-level cities. The study area is divided by terrain crossing GDP into four regions of Eastern developed, Central developing, Western underdeveloped, and Northwestern underdeveloped.

**Fig 1:**
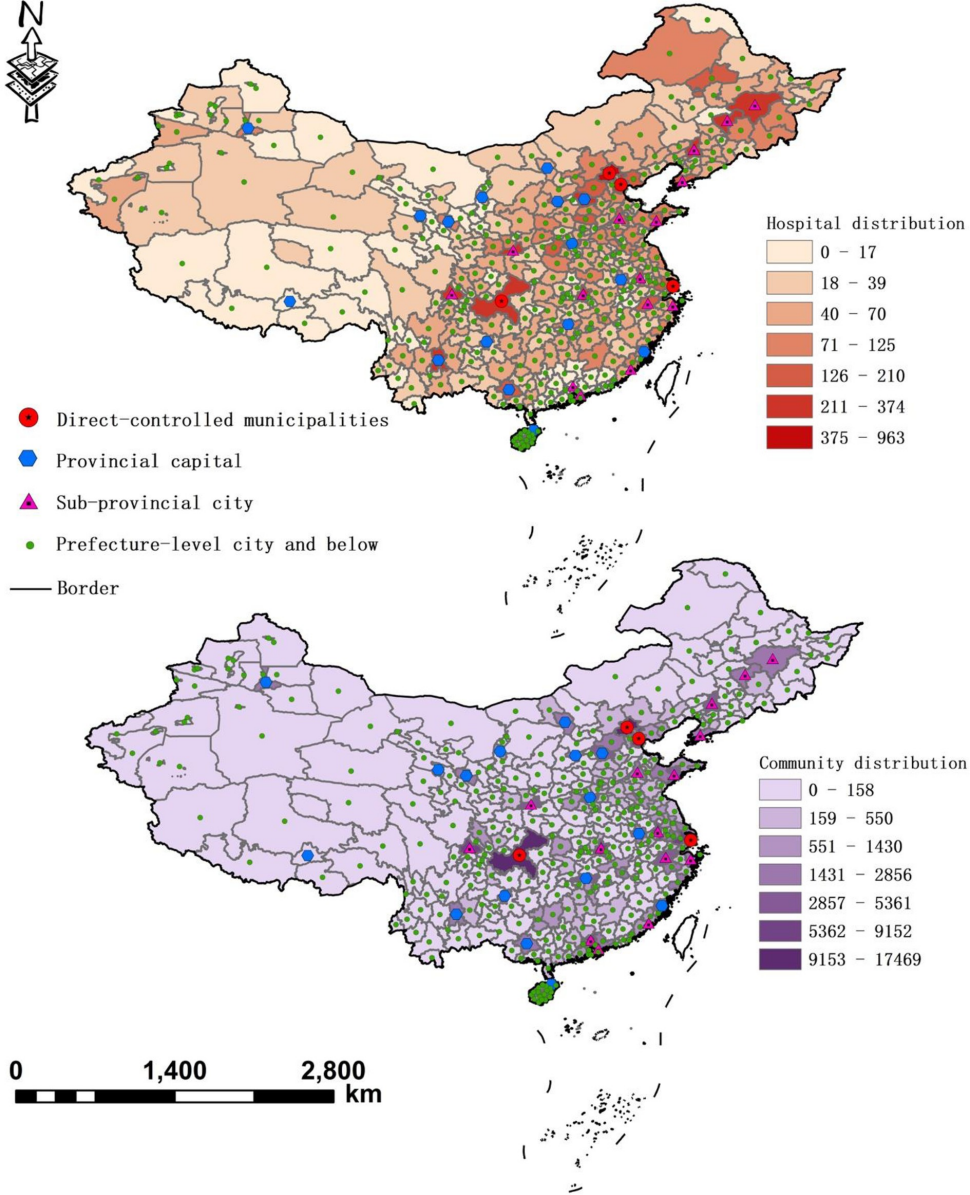
Spatial distributions of quality hospitals and housing estates within the study area.

Within the study area, we collected data on quality hospitals, populations and road networks and combined them in ArcGIS (version 10.4). Data sources include official statistical yearbooks and third-party websites. All the data are open sourced and our collection method complied with the terms and conditions for the website in each case. Fig 1 shows our study area and the integration of data, where the upper part presents the distribution of quality hospitals and the lower part shows the distribution of residential estates; and road networks are depicted as lines.

### Data

#### Geocoded inventory of quality hospitals

We started by assembling a geocoded inventory of quality hospitals scattered over the study area. We recognized hospital qualities following the current, most authoritative quality standard in China, namely, the Tertiary and Ten-Classes Hospital System (revised version 2011), issued by the National Health and Family Planning Commission. The system grades every hospital (both private and public hospitals) as tertiary, secondary or primary from high to low, according to the facility’s comprehensiveness, staff size, facilities, technological levels, equipment, managerial function, etc.; each tier is further scored as Class A (outstanding), B (fair) and C (qualified), while the tertiary tier has an additional grade of A^+^ (excellent), making a system of ten classes. Nonetheless, it might be dogmatical to assume only tertiary hospitals are quality hospitals, since primary and secondary hospitals, oriented toward community and multicommunity households, are playing increasingly important roles in strengthening the primary health care system in China [2]. Thus, in this research, we consider hospitals ranked higher than or equal to primary B in the specified ten-class system to be quality hospitals.

We first cross-referenced data from various sources to build a full list of city-specific quality hospitals. Data sources include China Health/Statistical Yearbooks, the NHC website (http://www.nhc.gov.cn/), websites of local health and family planning commissions, official and unofficial websites of hospitals such as <www.tjh.com.cn> (official) and <www.haodf.com> (unofficial). The numbers of physicians and beds of each hospital were also integrated into the list. We then registered each hospital with its longitude and latitude coordinates, which are available at Baidu Map <http://map.baidu.com/>. We also cross-checked the coordinates of each hospital with points of interest (POIs) that denote names, categories, addresses and coordinates of geographic entities extracted from Baidu Maps to ensure that a hospital is within its administrative boundaries and not located on water or offshore. Since the latest POI data were updated in 2017, all the data mentioned above are for 2016 to maintain the consistency of time of multisourced data. We finally rendered a geocoded inventory of 18,736 quality hospitals. The upper part of Fig 1 above shows their spatial distributions across different types of cities, presenting an intuitive impression of unequal distribution and a significant concentration of quality hospitals in direct-controlled municipalities. More detailed discussions will be introduced in the *Results and Discussions section*.

#### Population data

Statistical yearbooks in China only provide the population data best at the level of administrative division, lacking information on population distribution at the community level. Such a lack of information could be a serious problem for spatial analyses on health care accessibility since, as we mentioned above, communities (in China, a community usually consists of several housing estates) are basic service units and one of the most important factors for both the public and private sectors to consider when deciding whether to establish a hospital in a certain location. To capture the condition of demand as accurately as possible, metrics of population groups, consisting of their sizes (i.e., the numbers of households), wealth levels and geographic distributions, were synthesized from the fusion of multisourced data of various types, including POIs, data on population densities and housing features. All data is for the year 2017.

Computation of the community-level population followed a five-step process. First, geocoded housing estates in each city of our study area were identified by examining more than 22 million pieces of POIs, and a total of 231,079 estates were identified in this step (see the lower part of Fig 1. Second, for each estate, the number of blocks, the number of flats per block, and the average list prices are extracted from three major commercial websites of housing information in China, including <https://m.lianjia.com/>, <https://www1.fang.com/>, and <https://www.anjuke.com/>. We have taken the mean value when the average prices from different sources are different. Since the data on housing features is unavailable for some of the identified estates, we excluded them and finally managed to convert a dataset of 145,803 observations. Third, estates are divided into five classes by their average list prices in CNY, including Group 1 (<5,468), Group 2 (5,468–7,503), Group 3 (7,503–10,845), Group 4 (10,845–22,332), and Group 5 (>22,332). Noticeably, the list price normally exceeds the exact transaction price around 15% on average [25]; yet the list price does not lead to systematic variations on housing characteristics or community environments, which convey the main implication for residents’ wealth statuses. Thus, the use of list prices will not significantly violate our empirical analyses on the spatial accessibility across different wealth groups.

Fourth, the number of households in an estate was estimated by totaling all flats contained in each block. We did not consider the problem of vacancies because the available vacancy rate is generally calculated by dividing the subtotal of vacant flats over the total number of flats owned by city households [26]. Hence, adjusting the sum of households by vacancy rates would exclude the situation of rental occupancy, resulting in underestimates of demand conditions. Although our calculation may lead to an overestimate of the demand condition, the overestimate itself is not likely to interfere out final results about health care inequalities at the community level (reported in the last part of the *Results and Discussion section*) because, along with booming real estate prices in China, especially for upper-tier cities [26], opportunity costs of vacancies are also rising, and thus homeowners are increasingly less likely to keep their extra flats vacant instead of renting them out. However, if we do consider the vacancy rate of the host city, rental tenants as potential uses of health care services would be excluded and our final results are more likely to be biased.

**Fig 2:**
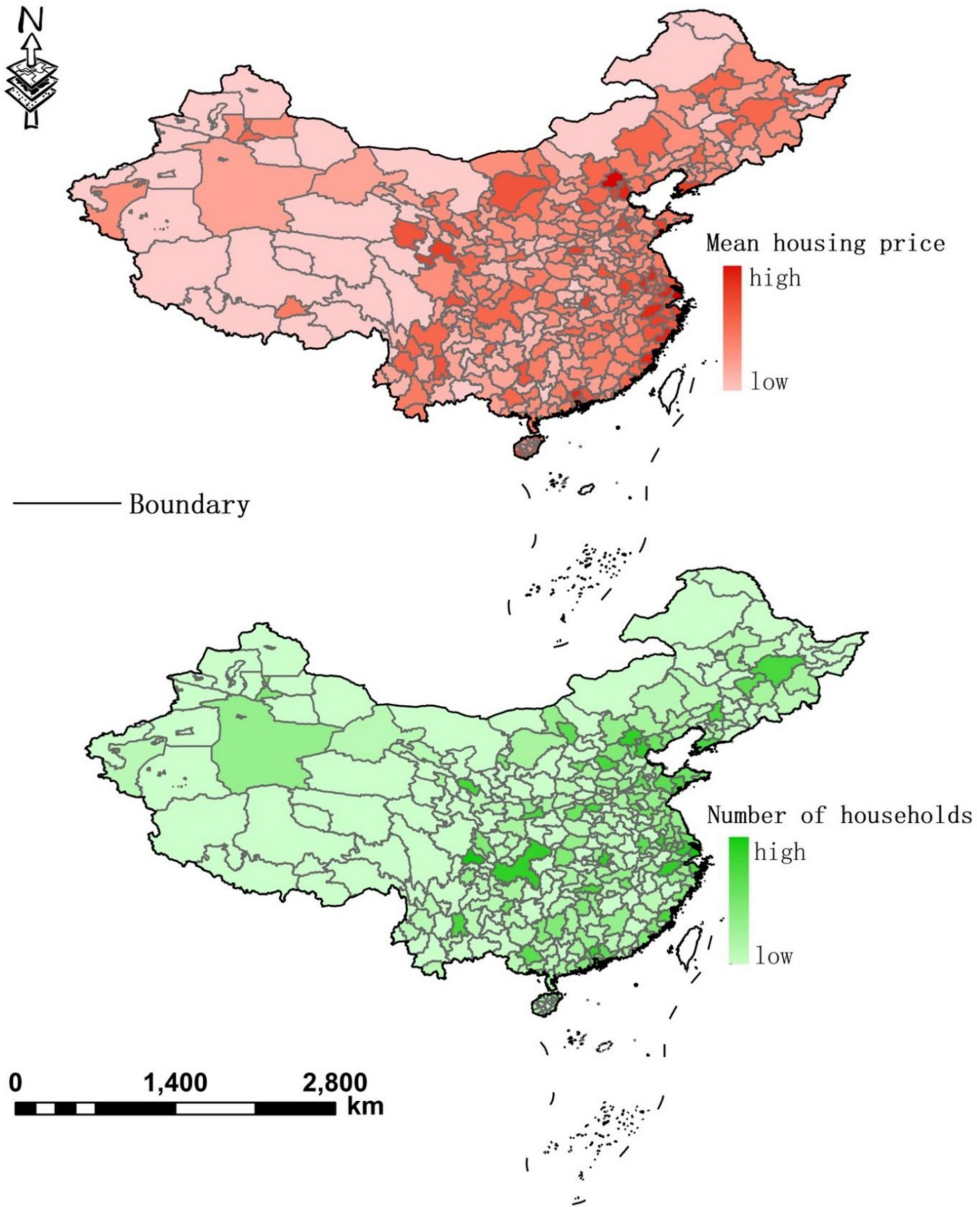
The matching image of the housing price layer and the household layer.

Through the above steps, we obtained the geographic distributions and the number of households of each wealth group by taking the average list price of an estate as the proxy for its residents’ wealth levels. This inference corresponded well with a priori evidence on the relationship between residents’ economic status and the market values of their properties [27, 28]. In the final step, we overlapped the housing price layer (at a 1-km spatial resolution) and the population density layer (sourced from LandScan2017^TM^) using GeoDa software and found that the spatial distribution of housing prices is significantly associated with that of household numbers (coeff. = 0.608), i.e., estates with higher list prices are also those with higher population densities (see Fig 2). This covariance is consistent with previous findings about the relation between population density and housing prices in urban China (e.g. [29]), which provides side-evidence supporting our computation of population. Table 1 presents the summary of statistics of our estimates on the mean prices of housing estates and the number of households in 20 major cities. We did not present statistical summaries of housing prices and household numbers in all sampled cities because of the limitation of page on the one hand, and on the other hand such a table would to a certain extent provide repetitive information with Figs 1 and 2. From Table 1 it can be seen that Beijing and Shanghai, the two most important metropolises in China, have the highest housing prices and the largest household numbers. While Shenzhen has similar housing prices with Shanghai, the number of households in the former is less than a half of that in the latter. Housing prices in cities such as Nanjing, Tianjin, Hangzhou and Xiamen are also striking, echoing previous findings of [28].

**Table 1:** Statistical summary of housing prices and household numbers in 20 major cities.

City	Mean housing prices (CNY/m^2^)	Number of households	City	Mean housing price (CNY/m^2^)	Number of households
Beijing	45,349	7,375,129	Hangzhou	18,168	2,014,805
Shanghai	43,431	5,317,392	Xi’an	6,496	2,432,508
Shenzhen	43,003	2,531,413	Jinan	10,575	944,702
Guangzhou	20,624	2,844,987	Ningbo	12,370	1,544,258
Tianjin	17,323	2,842,814	Dalian	10,467	1,835,533
Chongqing	7,076	4,202,342	Qingdao	12,679	1,014,060
Chengdu	8,020	3,403,871	Xiamen	30,863	321,329
Wuhan	10,546	2,196,256	Changchun	6,932	811,450
Changcha	6,695	2,037,318	Shenyang	7,406	1,337,619
Nanjing	21,582	2,060,887	Harbin	7,557	1,228,536

Source: Computed by the authors.

#### Road network

We extract the data on inner city road networks from OpenStreetMap, including national/provincial roads, primary/secondary/tertiary roads, and highways. We reconcile the data by connecting all road segments if they were not connected after digitization and deleting roads approaching bodies of water. The respective average travel speeds by vehicle on each type of road were obtained from the Highway Technical Standards of China, assigning 120 km/h to highways, 100 km/h to national roads, 80 km/h to provincial road, 60 km/h to primary roads, 50 km/h to secondary roads, and 30 km/h to tertiary roads. Walking speed was set as 5 km/h.

### Methods

#### Two-step floating catchment area (2SFCA)

Diverging the spatial accessibility of different wealth groups is one of the most widely used means to assessing health care inequalities. Furthermore, whether there is an adequate supply of services for targeted populations critically impacts the health care accessibility of individual households. In view of these, we adopted the 2SFCA method to measure health care accessibility and the supply‐to‐demand ratio for different wealth groups. The 2SFCA algorithm circumvents problems arising from the use of arbitrary administrative areas as reference frames, which, together with other merits such as distinct in concept and simple in calculation, make 2SFCA a typical and popular method to measure health care accessibility [11, 17].

The core of application of 2SFCA is the determination of ‘floating catchments’ for each demand site (*C*_*d*_) and supply site (*C*_*s*_), assuming that there is a geographic surface or threshold of travel time (*d*_0_) facing people who seek desired services and that people prefer nearer services to those farther away. Step 1 of 2SFCA computes *C*_*s*_ for hospital *j*, i.e., a supply site, as the reference frame for calculating the supply‐to‐demand ratio. The ratio is determined by the total service capacity of hospital *j* divided by the total demand volume, as illustrated in Eq (1)

$$V_{j}=\frac{S_{j}}{\sum_{k\epsilon d_{kj}\leq d_{0}}P_{k}}$$ (1)

where *V*_*j*_ denotes the supply-to-demand ratio, *S*_*j*_ the service capacity of hospital *j*, *d*_*kj*_ the travel time between location *k* and facility *j*, and *P*_*k*_ the population at location *k*.

Step 2 starts with considering patients at a demand site *i* searching for health care services that fall within their threshold travel time (*d*_0_). Here, we set *d*_0_ as one hour travel by vehicles, ranging from approximately 25 to 45 kilometers in different cities during rush hours according to *China Urban Traffic Report* (2019) [30]. We selected such a relatively high threshold because, as revealed by [3], hospitals in China are paid by both Social Health Insurance (SHI) and patient out-of-pocket payments, patients hence are inclined to search within a broad area to make sure that their SHI is accepted by the attended hospital. The area between services and *i* forms a catchment *i* ($C_{d}^{i}$); hospitals/services falling in $C_{d}^{i}$ are deemed to be accessible by that population. Calculating supply as the sum of accessible services offered by hospitals, geographical accessibility (*GA*_*i*_) at location *i* is expressed as:

$${GA}_{i}=\sum_{j\epsilon d_{ij}\leq d_{0}}V_{j}$$ (2)

where *d*_*ij*_ denotes the travel time between location *i* and hospital *j*. Catchments for different demand sites often overlap, suggesting that a hospital may fall into more than one *C*_*d*_, providing services for several demand sites. However, according to Eq 1, *S*_*j*_ is proportionately distributed among all in-reach demand sites, i.e., the calculation of *V*_*j*_ avoids the double counting problem. Thus, *GA*_*i*_ derived from Eq 2 can be considered as accounting for the location-dependent relationship between supply and demand.

#### Gini index

The Gini index (*GI*) is employed to identify any inequality existing in health care accessibility across groups with different wealth levels and population sizes on multiple scales (e.g., city, region, the entire nation). *GI* is a widely used indicator of inequality among values of a frequency distribution (e.g., households’ wealth levels, access to public services) within a nation or a certain population cluster [20, 31]. In this research, we analogize a community as a household, accessibility as household wealth levels, and measure inequality of health care accessibility according to Eq 3.

$$GI=1+\sum Y_{n}P_{n}-2\sum {\left(\sum P_{n}\right)}^{\prime }\;Y_{n}(n=1,\ldots ,5)$$ (3)

where *Y*_*n*_ denotes the ratio of averaged *GA*_*i*_ of category *n* to the overall averaged *GA*_*i*_ in a city (or region, nation), *P*_*n*_ is the ratio of the volume of category *n* to the total volume of communities (*TC*), and (∑P_i_)′ is equal to $\frac{\sum P_{i}}{TC}$. The value of *GI* varies between 0 and 1; when *GI* = 0 it expresses perfect equality with respect to health care accessibility per capita, while when *GI* = 1 it expresses maximal inequality among different values of health care accessibility per capita across different income group.

## Results and discussions

### Pro-developed distribution of health care provision

**Fig 3:**
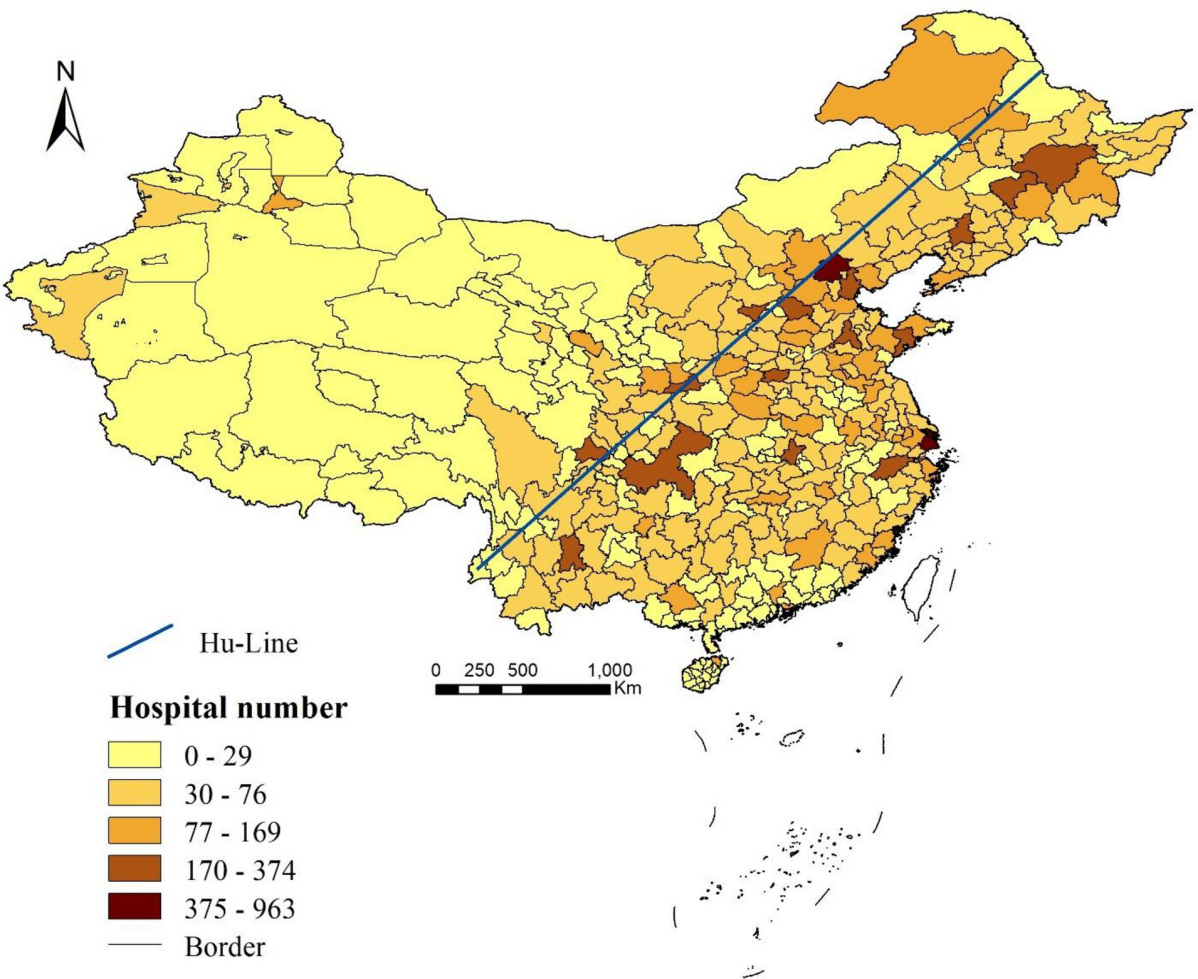
Distribution of quality hospitals (DoQ).

Putting the constraint of demand size aside, we first report results on the provision of quality health care services before getting into a fuller analysis (see Fig 3). At a national scale, we identify, with little surprise, pro-developed inequalities in the distribution of quality hospitals (hereafter abbreviated as DoQ). The Gini index for DoQ across the entire nation is 0.745, echoing previous findings such as [24, 32]. On a city scale, the results show that quality hospitals visibly agglomerate into major municipalities and capital cities, demonstrating a pattern of decreasing supply along with the degradation of cities’ administrative rankings: in a provincial city, the average number of quality hospitals is 598, in a subprovincial city, 193, a capital city, 121, and in a prefecture-level city, there are only 34 quality hospitals on average.

As shown in Fig 3, cities with more quality hospitals are almost exclusively southeast of the Hu Line, while cities with fewer quality hospitals are mostly located in the Northwest, especially the Western region, which is among the lowest echelons of the GDP ranking [32]. The Hu Line is a famous geographical line proposed by Hu Huanyong in 1935 to mark the striking difference in China’s population and economies. Thus, the above results further indicate that after 80 years of urbanization and development, the Hu Line might be endowed with a wider connotation that points out a serious problem facing an economically vulnerable population, a less densely inhabited Northwestern area where quality health care services are remarkably lacking.

Regarding within-group inequalities, we also identify both within-region and within-city disparities in DQ. The Gini indices of the Eastern, Central, Northeastern, and Western regions are 0.687, 0.708, 0.750, and 0.771, respectively. Compared with the national Gini index (= 0.745), unevenness in the Northeastern and Western regions obviously contributes large proportions to the overall inequalities, possibly because these two regions cover many underdeveloped areas, while they also embrace sub/provincial cities, such as Chongqing, Chengdu, Changchun, and Harbin, which are much more developed and equipped with more abundant quality health care services.

### Unexpected patterns of accessibility considering demand on multiple scales

We then compute the geographic accessibility of quality health care provision subject to different supply-demand conditions (hereafter abbreviated as GAPSD) and examine whether the spatial distribution of GAPSD is unequal using multiple scales. GAPSD are derived from Eq (2), where *V*_*i*_ is calculated by the number of households at the community-level in a catchment area. A higher value of GAPSD indicates more accessible health care services for individual households living in a certain area (region/city/community). The GAPSD results in 31 provinces, major cities, regions, and economic zones are presented in Table 2. Nationally, there are still significant disparities in GAPSD as expressed by a high Gini index (= 0.737). The unexpected aspect of the derived results, however, is that the “pro-developed” inequalities in DoQ as identified above seem to have been inverted; the relationship between the level of development and GAPSD in many areas has turned toward “pro-underdeveloped”.

**Table 2:** GAPSD in provinces, major cities, and regions.

Provinces	GAPSD	Subprovincial cities	GAPSD	Prefecture cities	GAPSD (Top 10)	Regions	GAPSD
1-Guangdong	4.252	Shenzhen	2.782	Jiamusi	4976.1	Eastern	7.143
2-Jiangsu	7.183	Guangzhou	4.211	Jinchang	4649.78	Central	18.441
3-Shandong	13.718	Chengdu	4.608	Shiyan	3510.98	Western	10.726
4-Zhejiang	14.068	Wuhan	7.372	Fuzhou	2638.98	Northeastern	15.558
5-Henan	19.420	Hangzhou	6.607	Jingzhou	1833.685		
6-Sichuan	6.196	Nanjing	6.462	Datong	1788.95		
7-Hubei	16.122	Ningbo	8.575	Yichun	729.296		
8-Hunan	15.989	Qingdao	7.167	Jian	657.99		
9-Hebei	13.089	Jinan	12.888	Xinganmeng	628.147		
10-Fujian	12.626	Xi’an	5.861	Yaan	517.549		
**11-Shanghai**	7.467	Dalian	3.480				
**12-Beijing**	4.659	Shenyang	9.329				
13-Anhui	20.528	Xiamen	14.106				
14-Liaoning	11.372	Changchun	11.641				
15-Shaanxi	10.174	Harbin	6.744				
16-Jiangxi	22.294	**Average**	**7.404**	**Average**	**138.792**		
**17-Chongqing**	9.581						
18-Guangxi	18.249						
**19-Tianjin**	4.505						
20-Yunnan	15.140						
21-Inner-Mongolia	15.971		Chart:				
22-Shanxi	24.482		Eastern region consists of provinces no. 1, 2, 3, 4, 9, 10, 11, 12, 18,				
23-Heilongjiang	20.602		19 and 28				
24-Jilin	17.759		Central region consists of provinces no. 5, 7, 8, 13, 16 and 21				
25-Guizhou	17.234		Western region consists of provinces no. 6, 15, 17, 20, 22, 25, 26,				
26-Xinjiang	13.935		27, 29, 30 and 31				
27-Gansu	17.286		Northeastern consists of provinces no. 14, 23, and 24				
28-Hainan	6.255						
29-Ningxia	13.386						
30-Qinghai	29.805						
**Average of provincial cities**		**6.553**					

The first column of Table 2 presents the GAPSD values of 30 provinces or provincial cities. For economically developed provinces, such as Guangdong, Jiangsu and Sichuan, the GAPSD values are only 4.252, 7.183 and 6.196, respectively, which are much lower than those of underdeveloped provinces such as Qinghai, Ningxia and Guizhou, for which the GAPSD values are 13.935, 13.386 and 29.805, respectively. For the four provincial cities, i.e., Beijing, Shanghai, Chongqing and Tianjin, the average GAPSD is as low as 6.553, while that in prefecture-level cities (shown in the third column of Table 3) is 138.792, surprisingly more than twenty times higher than the former. For the fifteen provincial cities, including international metropolises such as Shenzhen (GAPSD = 2.782) and Guangzhou (GAPSD = 4.211), the average GAPSD is also significantly low (7.404) compared with other capital cities (GAPSD = 18.762 on average), although it is higher than that of the four provincial cities.

**Table 3:** Top 10 cities with the most even and uneven distribution of GAPSD.

	The most uneven GAPSD			The most even GAPSD		
Rank	City	Level of Admin.	GI	City	Level of Admin.	GI
1	Zhaoqing	Prefecture	0.799	Xinyu	Prefecture	0.0028
2	Jiangmen	Prefecture	0.663	Bengbu	Prefecture	0.033
3	Huizhou	Prefecture	0.618	Liuzhou	Prefecture	0.048
4	Dongguan	Prefecture	0.594	Hohhot	Capital	0.048
5	Bayingolin Mongol	Autonomous Prefecture	0.528	Zhuzhou	Prefecture	0.053
6	Deyang	Prefecture	0.518	Qionghai	Prefecture	0.063
7	Xiangyang	Prefecture	0.513	Jilin	Prefecture	0.066
8	Xianyang	Prefecture	0.464	Haikuo	Capital	0.072
9	Zhangjiakou	Prefecture	0.444	Yinchuan	Capital	0.072
10	Shaoxing	Prefecture	0.424	Harbin	Capital	0.073

Scaling-up to situations in zones and regions, the Eastern region is most developed and has the lowest GAPSD (= 7.143), followed by the Western (= 10.726), Northeastern (= 15.558) and Central (= 18.441) regions, showing uncertain relationships between the level of development and GAPSD. A candidate reason behind the inverted pattern of “low GAPSD in developed areas” is very likely to be the high population densities in these areas. This reason may be simple and direct; however, surprisingly, such a pattern itself has not been revealed in detail in previous studies.

Focusing on within-region/city inequalities, we find that the Eastern region, with the lowest GAPSD, has the most even distribution of services (GI = 0.658), while the Northeastern region, in which the provision of quality health care services is scant, has the most uneven distribution of GAPSD (GI = 0.776). Within cities, the top 10 with the most uneven GAPSDs are presented in Table 3. As shown in the column on the left, the four cities at the top of the uneven GAPSD echelon are small prefecture-level cities located in the Guangdong-Hong Kong-Macao Greater Bay Area. This is possibly because the four cities are all adjacent to two metropolises, Guangzhou and Shenzhen; in this case, quality hospitals may well agglomerate in border areas that are closer to those developed cities. In other words, the location character of the four small cities makes them suffer from an “agglomeration shadow” imposed by neighboring metropolises regarding the provision of quality health care services. Such uneven distribution highlights the importance of quality health care provision in small or less developed cities surrounding developed ones.

As shown in the right column of Table 3, four cities with both relatively abundant quality hospitals and an even distribution of GAPSD stand out, which are capital cities of Hohhot, Haikou, Yinchuan, and Harbin. Among provincial cities, Chongqing has the most uneven distribution of GAPSD (GI = 0.416), while the Gini indices for Beijing, Shanghai and Tianjin are as low as 0.170, 0.112 and 0.169, respectively, possibly because Chongqing has the broadest area and the most complex landform among the four.

### Service-poor “rich” communities vs. service-rich “poor” communities

Zooming to GAPSD at a finer granularity, we computed the average GAPSD for each of the five categories of residential community (see Section *Materials and Methods*) on multiple scales. Intuitively, we discerned an inverse relation between GAPSD and the average list price of a house in the community. Since the community rate can reflect its residents’ wealth level, as illustrated above, the diverging GAPSD for different rate categories could also be interpreted as that for different wealth groups. Across the nation, the relationship forms an “inverted pyramid” structure (see Table 4), i.e., the “richest” communities have the lowest GAPSD (= 5.220), while the “poorest” communities have the highest GAPSD (= 19.055). The gap here is 3.65 (= 19.055/5.219), representing the average level of inequality between wealth groups/communities across the country.

**Table 4:** GAPSD for different wealth groups/communities nationwide.

Wealth groups/communities	GAPSD
<5,468	19.055
[5,468, 7,503]	11.291
[7,503, 10,845]	7.507
[10,845, 22,332]	6.114
>, 22,332	5.220

**Fig 4:**
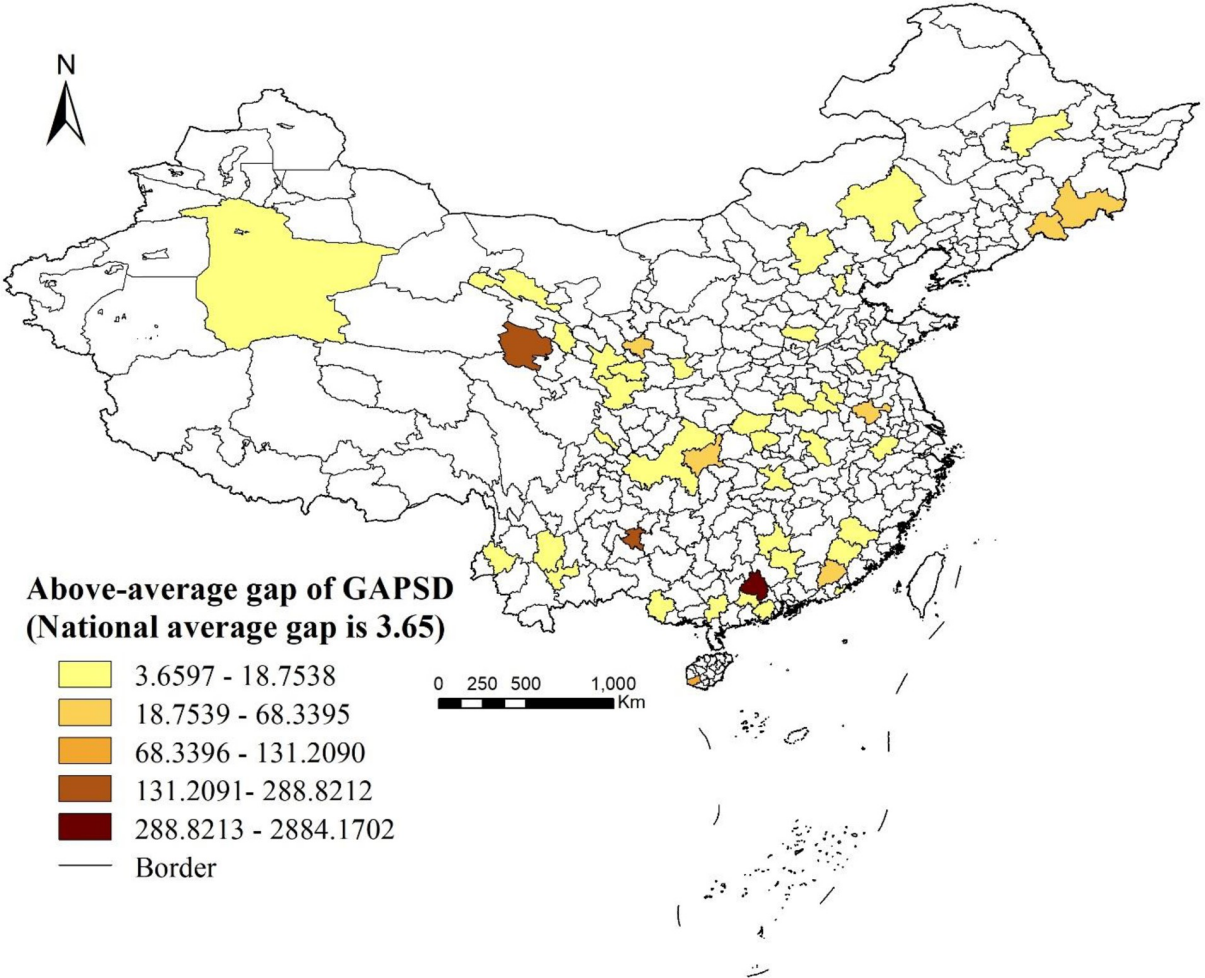
Distribution of cities with an above-average gap of GAPSDs.

The results for the 19 provincial and subprovincial cities are presented in Table 5, which again show an inverse relation between GAPSD and rates of residential communities. The average GAPSD in these 19 cities is 7.225. At two extremes of the spectrum are 10.507 for communities at the bottom deciles in Chongqing and 2.582 for communities at the top decile in the same city. Within cities, we find 46 (out of 361) have gaps above the national average (= 3.65), among which Chuzhou in Anhui Province, Baishan in Jilin Province, Anshun in Guizhou Province, and Zhaoqing in Guangdong Province have gaps as great as over 50 (see Fig 4).

**Table 5:** GAPSD for different wealth groups/communities at a national level and in 19 cities.

GAPSD/Scale	Group 1 (<5,468)	Group 2 ([5,468, 7,503])	Group 3 ([7,503, 10,845])	Group 4 ([10,845, 22,332])	Group 5 (>22332)
**National-wide**	19.055	11.290	7.507	6.114	5.219
Shanghai	6.396	6.711	7.637	8.146	8.627
Beijing	4.384	3.705	4.559	4.968	5.680
Tianjin	4.154	3.705	4.416	4.869	5.194
Chongqing	10.507	4.234	3.001	2.606	2.582
Shenzhen	0.142	0.159	0.207	0.213	0.183
Guangzhou	0.082	0.167	0.206	0.223	0.229
Chengdu	3.715	4.137	5.005	5.154	5.031
Wuhan	5.521	7.070	7.811	8.203	8.250
Hangzhou	6.666	5.911	6.535	6.892	7.031
Nanjing	5.988	5.963	6.411	6.787	7.162
Ningbo	9.752	8.886	8.341	8.055	7.848
Qingdao	8.131	5.680	6.576	7.892	7.555
Jinan	21.405	10.324	10.737	10.926	11.083
Xi’an	5.872	5.724	5.748	5.925	6.037
Dalian	4.104	2.638	3.259	3.633	3.764
Shenyang	8.508	8.591	9.341	10.030	10.174
Xiamen	11.384	12.881	14.988	15.519	15.759
Changchun	12.810	11.178	11.343	11.341	11.534
Harbin	6.049	6.798	6.783	7.161	6.927

To rigorously examine the inverse relation between GAPSD and wealth level, we employ dummy variables representing the specified five groups of housing prices (proxies for the wealth levels of community residents) and regress them on the corresponding average GAPSD using the Ordinarily Linear Regression (OLS) Method. Specifically, we use [0,0,0,0] to denote Group 1 (i.e., the reference group), [1,0,0,0] for Group 2, [0,1,0,0] for Group 3, [0,0,1,0] for Group 4, and [0,0,0,1] for Group 5. Regression results are provided in Column I~V of Table 6, where in Column I are the coefficients at the national level and in Column II~V are those for the four different regions. Using the same method, we also compare the coefficients of GAPSD against housing prices (Column VI of Table 6) with those derived by replacing GAPSD with spatial accessibility (Column VII of Table 6). Spatial accessibility is calculated based on $V_{j}=\frac{S_{j}}{\sum_{k\epsilon d_{kj}\leq d_{0}}P_{k}}$ (Eq [1]), which indicates the accessibility of heal care in the pure spatial sense, i.e., the availability of health care services in a catchment area without considering the number of potential users they serve.

**Table 6:** Results of GAPSD against different wealth groups/communities and comparison between GAPSD and spatial accessibility regarding their correlations with housing prices.

	(I)	(II)	(III)	(IV)	(V)	(VI)	(VII)
Coeff.	GAPSD_1	GAPSD_1 in Eastern developed	GAPSD_1 in Central developing	GAPSD_1 in Western underdeveloped	GAPSD_1 in Northeastern underdeveloped	GAPSD_2	Spatial accessibility
Housing price/ Group 1	-0.000091*** (-7.423)						0.000079*** (98.156)
Housing price/ Group 2	-0.00014*** (-8.558)	-3.970*** (-13.592)	-15.191*** (-6.552)	-6.032*** (-3.895)	-9.190 (-1.281)	-7.760*** (-10.074)	0.000175*** (141.694)
Housing price/ Group 3	-0.000118*** (-9.600)	-5.203*** (-17.883)	-22.380*** (-10.323)	-9.648*** (-6.227)	-18.947** (-2.448)	-11.544*** (-14.983)	0.000222*** (153.966)
Housing price/ Group 4	-0.000143*** (-8.557)	-7.177*** (-27.868)	-23.220*** (-9.830)	-8.619*** (-3.319)	-20.765** (-2.097)	-12.937*** (-16.763)	0.000248*** (156.537)
Housing price/ Group 5	-0.000147*** (-12.105)	-7.807*** (-31.494)	-1.434 (-.166)	-7.350 (-.866)	-17.851 (-.568)	-13.832*** (-17.941)	0.000261*** (152.280)
*R* ^*2*^	0.041					0.288	0.456
Observation							145,803

Notes: (1) Model (I~V): *GAPSD*_*i*_ = *c*+*β*_*i*_*Dummy*_*i*_+*ε*_*i*_, Model VI: *GAPSD*_*i*_ = *c*+*β*_*i*_*Housing Price*_*i*,*iϵGoupj*_+*ε*_*i*_, Model VII: *Spatial accessbility*_*i*_ = *c*+*β*_*i*_*Housing Price*_*i*,*iϵGoupj*_+*ε*_*i*_; (2) t-value in parentheses; (3) *** and ** indicate significance levels of 1 and 5%, respectively.

By and large, we find a significant inverse relation between GAPSD and housing price ranks, implying an “abnormal” fact that it is those among the richest who are deprived groups in the sense of quality health care services per capita. This phenomenon is especially notable in the Eastern region (Column II), in which all four coefficients highly significant at the 1% level, and the absolute values of the adverse edge effect in wealth/rate progressively rise as the horizontal edge distance gradually increases. Implicitly, the provision of quality health care services in the Eastern region, as abundant as they are, still barely catch up the agglomeration of population in high-price communities that are generally located in desirable neighborhoods in cities. Comparatively, in the other three regions (Column III~V), we find that the inverse relation between GAPSD and wealth level is insignificant for Group 5, while in the Northeastern region, the wealth level of Group 2 is also irrelevant. Such a pattern points out another major issue in underdeveloped regions: perhaps only middle-high- to low-wealth groups’ individual accessibility to quality health care is affected by their population sizes, while for individual households in the top wealth group, health care accessibility is not affected by the potentially high demand. Candidate reasons include that health care services targeting high-wealth communities in underdeveloped regions might be located mainly in areas with an “access threshold” imposed by high housing prices, or population densities of high-wealth communities, unlike those in the Eastern region, are actually lower than middle-high and low-wealth communities, i.e., the relationship between housing prices and population density in underdeveloped regions largely exhibits an inverted “U” shape. From Column VII we can see that the spatial accessibility significantly increases along with increasing housing prices. After considering the demand, however, the accessibility of health care (Column VI) consistently turn to negative for all housing estates with average prices falling in the specified five groups. Combining Column VI and Column VII, it reveals that the consideration of the demand side plays an important role in the analyses of health care inequalities in China.

## Concluding remarks

Substantial improvements in promoting health care equality after the transformative reform in 2009 were affirmed by various quarters [33]. However, there are indications that the health care distribution has been continuously uneven and that evidence of pro-rich inequities. Such inconsistencies have been voiced in parallel with another policy issued in 2012, which implicitly invites private provision of health care services and is deemed to be weakening the effects of the 2009 reform. Subject to such an anfractuous situation, a means of resolving these issues could be, as we have done in this research, to reveal the state of health care inequality and to examine whether individuals at varying wealth levels could access equal and adequate quality care under the current health care distribution.

Translating health care inequality into spatial principles is rather challenging. Among a great deal of approaches to this topic, spatial accessibility is a classic measure for assessing inequality in health care delivery [9, 11, 16]. A pithy line of reasoning, indicated by seminal works, such as [9, 10, 17, 34], helps rationalize the role of spatial accessibility in health care inequality. Specifically, price-based rationing has long been recognized as a major barrier to equality; thus, equality is considered a political philosophy associated with market intervention [35, 36]. Subject to forces from both sides, geographical configurations of health care services are primarily determined by individual providers constrained by both the law of market competition (i.e., diversified services made available to prospective users with varying demand characteristics) and political regulation in an egalitarian spirit (i.e., treating all citizens equally according to their needs). In this sense, spatial accessibility can be considered a hybrid result of the supply-demand interaction under simultaneous influences of efficiency-based rationing and equality-based intervening and thereby virtually reflects the overall solidarity and performance of equality. A typical example is that geographical differentiations in supply would make different groups face different travel and time costs for using the same services, which would consequently produce disparities in accessibility and diverging patterns of utilization and health outcomes [37–39].

Moreover, spatial accessibility must be considered under location-dependent supply-demand conditions. The supply-demand conditions might exacerbate existing unequal accessibility prevalent in communities in cases of long distances to hospitals together with inadequate services available for targeted populations [17]. In some other cases, by contrast, the supply-demand conditions may well invert the intuitive impression of pro-rich inequality when high-wealth communities close to hospitals have high population densities, which effectively deprive residents’ per capita opportunity for quality care. Thus, only by interpreting spatial accessibility in the specific contexts of supply and demand would such knowledge provide an effective tool and springboard for revealing the spatial organization of health care, for investigating its relation with health care utilization and outcomes, and, more importantly, for revealing underlying reasons for inequalities that may fuel exploration of how improvements can be made [9, 14, 17, 34].

Our results hinge on two findings. On the one hand, quality health care services are seriously concentrated in cities with high administrative rankings and development levels. This pattern of unevenness is evidenced by remarkable facts that major cities display absolute advantages in the provision of quality health care services, while fewer quality hospitals are located in prefecture-level cities. The same pattern is also reflected in the dominant contribution of within-group disparities in the Northeast and Western regions to overall inequality nationwide. Trends of huge disparities within underdeveloped regions and provinces indicate eminent pro-developed inequality and a serious problem of unequal allocation of quality health care resources.

On the other hand, we discern “pro-poor/underdeveloped” inequality in accessibility to health care after accounting for the size of the population each facility serves (GAPSD). Specifically, we find that GAPSD tends to decrease along with increases in administrative ranking s of cities and, with a finer granularity, in wealth ratings of communities (and thereof residents’ wealth levels). Cities from the prefecture level to the provincial level have sharply decreasing GAPSD, from 138.792 to 6.553. Across the country, the “richest” communities (rates >22,332) have the lowest per capita accessibility to quality care (GAPSD = 5.220), while the “poorest” communities (rates <5,468) have the greatest per capita accessibility to quality care (GAPSD = 19.055). Notably, we have shown that in the Eastern region, the relatively abundant quality health care adjacent to high-price communities is still inadequate with respect to population densities. More eminently, we find that in underdeveloped regions, the most expensive communities (and thereof groups at the top wealth deciles) tend to have more access to quality care, not only because the provision is abundant in these neighborhoods but also because the demand may be restricted by high housing prices, thus giving rise to a more serious problem of unevenness.

This research makes methodological contributions to the literature. Our approach to the population size of wealth groups (communities) has successfully revealed an “unexpected” pattern of health care accessibility, while pro-rich/developed inequalities have been the dominant voice in the literature. The deficiency of previous approaches, rather than a neglect of the demand constraint in previous studies, is likely to be an inaccurate capture of the demand volume instead. Some studies resort to the actual use of health care services, which is indeed a very precise description of demand but would only produce *ex post* evidence about inequalities, possibly failing to offer a basis for identifying vulnerabilities in, or making predictions about, capacities for addressing public health emergencies such as the COVID-2019 outbreak.

This research is also of policy relevance. For relatively developed areas in urban China, our findings suggest that the concentration of quality health care services in more developed cities and regions or ‘richer’ communities within a city—a fruit of forces from both market competition and policy intervention—does not necessarily exacerbate inequalities subject to increasing population pressure and the quest for a better life in urban China. This seemingly counterintuitive finding actually may not surprise urban researchers after accounting for the unique pattern of population distribution in China over the past four decades. It has been reported that population in China has continuously agglomerated into prosperous cities [40]; in those prosperous cities, meanwhile, the development of downtown areas has been facing great pressures of population concentration [41]. In this case, it might be both effective and equal for the government to enhance, at least to pay attention to, the supply of health care services in ‘rich’ cities and communities. An even distribution of health care services in the pure spatial sense may be just a kind of superficial equality. Comparatively, for underdeveloped cities and regions, the implication is that gaps between housing prices (wealth levels) have fostered more severe health care inequalities compared to those in developed areas, which calls for innovations in policies to encourage health care provision and paying attention to a social production of “wealth threshold” for access to quality health care. In other words, the current problem of health care inequalities might be more severe within underdeveloped regions instead of between regions.

Finally, we recognize the limitations of our investigation, including the rough identification of health care qualities, a lack of consideration of primary health care and clinic services, and the separation of private/public hospitals for comparative analysis. In addition, a city-specific division of housing price groups (proxy of wealth levels) may enhance the rigorousness and the potential contribution of this research. Thus, this research can be improved by compensating for these limitations. Another direction for future research could be shifting the focus on large-scale analysis to a relatively “micro” angle for acquiring more detailed information on the demand side.

## References

[1] Gao J, Tang S, Tolhurst R, Rao K. Changing access to health services in urban China: implications for equity. Health Policy and Planning. 2001; 16(3): 302–312. doi: 10.1093/heapol/16.3.302 11527871

[2] Li L, Fu H. China’s health care system reform: Progress and prospects. The International Journal of Health Planning and Management. 2017; 32(3): 240–253. doi: 10.1002/hpm.2424 28612498

[3] Yip W, Hsiao W. Harnessing the privatisation of China’s fragmented health-care delivery. The Lancet. 2014; 384(9945): 805–818. doi: 10.1016/S0140-6736(14)61120-X 25176551 PMC7159287

[4] Yip W, Fu H, Chen AT, Zhai T, Jian W, Xu R, et al. (2019). 10 years of health-care reform in China: progress and gaps in universal health coverage. The Lancet. 2019; 394(10204): 1192–1204. doi: 10.1016/S0140-6736(19)32136-1 31571602

[5] Wang X, Yang H, Duan Z, Pan J. Spatial accessibility of primary health care in China: a case study in Sichuan Province. Social Science & Medicine. 2018; 209: 14–24. doi: 10.1016/j.socscimed.2018.05.023 29778934

[6] Marten R, McIntyre D, Travassos C, Shishkin S, Longde W, Reddy S, et al. An assessment of progress towards universal health coverage in Brazil, Russia, India, China, and South Africa (BRICS). The Lancet. 2014; 384(9960): 2164–2171. 10.1016/S0140-6736(14)60075-1 PMC7134989 24793339

[7] Myint CY, Pavlova M, Thein KNN, Groot W. A systematic review of the health-financing mechanisms in the Association of Southeast Asian Nations countries and the People’s Republic of China: Lessons for the move towards universal health coverage. PloS One. 2019; 14(6): e0217278. doi: 10.1371/journal.pone.0217278 31199815 PMC6568396

[8] Aday LA, Andersen R. A framework for the study of access to medical care. Health Services Research. 1974; 9(3): 208–220. 4436074 PMC1071804

[9] Guagliardo MF. Spatial accessibility of primary care: concepts, methods and challenges. International Journal of Health Geographics. 2004; 3(1): 3. doi: 10.1186/1476-072X-3-3 14987337 PMC394340

[10] Andersen RM, Davidson PL, Baumeister SE. Improving access to care. Changing the US health care system: key issues in health services policy and management. San Francisco: Jossey-Bass. 2014; 36(3): 33–69.

[11] Langford M, Higgs G, Fry R. Multi-modal two-step floating catchment area analysis of primary health care accessibility. Health & Place. 2016; 38: 70–81. doi: 10.1016/j.healthplace.2015.11.007 26798964

[12] Higgs G, Langford M, Jarvis P, Page N, Richards J, Fry R. Using Geographic Information Systems to investigate variations in accessibility to ‘extended hours’ primary healthcare provision. Health & Social Care in the Community. 2019; 27(4): 1074–1084. doi: 10.1111/hsc.12724 30723952

[13] Guo Y, Chang SS, Chen M, Yip PS. Do poorer areas have poorer access to services in Hong Kong? A small-area analysis based on multiple spatial accessibility indicators. Social Indicators Research. 2018; 138(1): 1–21.

[14] Naylor KB, Tootoo J, Yakusheva O, Shipman SA, Bynum JP, Davis MA. Geographic variation in spatial accessibility of US healthcare providers. PloS One. 2019; 14(4): e0215016. doi: 10.1371/journal.pone.0215016 30964933 PMC6456202

[15] Ghorbanzadeh M, Kim K, Ozguven EE, Horner MW. A comparative analysis of transportation-based accessibility to mental health services. Transportation Research Part D: Transport and Environment. 2020; 81: 102278.

[16] Gulliford M, Figueroa-Munoz J, Morgan M, Hughes D, Gibson B, Beech R, et al. What does’ access to health care mean?. Journal of Health Services Research & Policy. 2002; 7(3): 186–188. 12171751 10.1258/135581902760082517

[17] Avdic D. Improving efficiency or impairing access? Health care consolidation and quality of care: Evidence from emergency hospital closures in Sweden. Journal of Health Economics. 2016; 48: 44–60. doi: 10.1016/j.jhealeco.2016.02.002 27060525

[18] Sun J, Luo H. Evaluation on equality and efficiency of health resources allocation and health services utilization in China. International Journal for Equity in Health. 2017; 16(1): 127. doi: 10.1186/s12939-017-0614-y 28709422 PMC5513103

[19] Jin Y, Zhu W, Yuan B, Meng Q. Impact of health workforce availability on health care seeking behavior of patients with diabetes mellitus in China. International Journal for Equity in Health. 2017; 16(1): 80. doi: 10.1186/s12939-017-0576-0 28666449 PMC5493891

[20] Li C, Dou L, Wang H, Jing S, Yin A. Horizontal inequity in health care utilization among the middle-aged and elderly in China. International Journal of Environmental Research and Public Health. 2017; 14(8): 842. doi: 10.3390/ijerph14080842 28933772 PMC5580546

[21] Tao Z, Cheng Y, Zheng Q, Li G. Measuring spatial accessibility to healthcare services with constraint of administrative boundary: A case study of Yanqing District, Beijing, China. International Journal for Equity in Health. 2018a; 17(1): 1–12. doi: 10.1186/s12939-017-0710-z 29334979 PMC5769485

[22] Tao Z, Yao Z, Kong H, Duan F, Li G. Spatial accessibility to healthcare services in Shenzhen, China: Improving the multi-modal two-step floating catchment area method by estimating travel time via online map APIs. BMC Health Services Research. 2018b; 18(1): 1–10. doi: 10.1186/s12913-017-2770-6 29743111 PMC5944163

[23] Lu C, Zhang Z, Lan X. Impact of China’s referral reform on the equity and spatial accessibility of healthcare resources: A case study of Beijing. Social Science & Medicine. 2019; 235: 112386. doi: 10.1016/j.socscimed.2019.112386 31272079

[24] Yu M, He S, Wu D, Zhu H, Webster C. Examining the multi-scalar unevenness of high-quality healthcare resources distribution in China. International Journal of Environmental Research and Public Health. 2019; 16(16): 2813. doi: 10.3390/ijerph16162813 31394765 PMC6720903

[25] Dinkel M, Kurzrock BM. Asking prices and sale prices of owner-occupied houses in rural regions of Germany. Journal of Interdisciplinary Property Research. 2012; 13(1): 5–25.

[26] Glaeser E, Huang W, Ma Y, Shleifer A. A real estate boom with Chinese characteristics. Journal of Economic Perspectives. 2017; 31(1): 93–116.

[27] Ross CE, Mirowsky J. Neighborhood socioeconomic status and health: context or composition?. City & Community. 2008; 7(2): 163–179.

[28] Wang Y, Wang S, Li G, Zhang H, Jin L, Su Y, et al. Identifying the determinants of housing prices in China using spatial regression and the geographical detector technique. Applied Geography. 2017; 79: 26–36.

[29] Zhou Q, Shao Q, Zhang X, Chen J. Do housing prices promote total factor productivity? Evidence from spatial panel data models in explaining the mediating role of population density. Land Use Policy. 2020; 91: 104410.

[30] Intelligent Transportation Joint Lab. <China Urban Traffic Report (2019)>. https://huiyan.baidu.com/cms/report/2019annualtrafficreport/. 2020 (Accessed in May, 2020).

[31] Wu J, Chen H, Wang H, He Q, Zhou K. Will the opening community policy improve the equity of green accessibility and in what ways?—Response based on a 2-step floating catchment area method and genetic algorithm. Journal of Cleaner Production, 2020; 263: 121454.

[32] Yin C, He Q, Liu Y, Chen W, Gao Y. Inequality of public health and its role in spatial accessibility to medical facilities in China. Applied Geography. 2018; 92: 50–62.

[33] Yip W, Hafez R. Reforms for improving the efficiency of health systems: lessons from 10 country cases. World Health Organization. 2015.

[34] McLafferty SL. GIS and health care. Annual Review of Public Health. 2003; 24(1): 25–42. doi: 10.1146/annurev.publhealth.24.012902.141012 12668754

[35] Cutler DM. Equality, efficiency, and market fundamentals: the dynamics of international medical-care reform. Journal of Economic Literature. 2002; 40(3): 881–906.

[36] Measurement Wang F., optimization, and impact of health care accessibility: a methodological review. Annals of the Association of American Geographers. 2012; 102(5): 1104–1112. doi: 10.1080/00045608.2012.657146 23335813 PMC3547595

[37] Le Grand J. Equality and choice in public services. Social Research: An International Quarterly. 2006; 73(2): 695–710.

[38] Davy C, Harfield S, McArthur A, Munn Z, Brown A. (2016). Access to primary health care services for Indigenous peoples: A framework synthesis. International Journal for Equity in Health. 2016; 15(1): 163. doi: 10.1186/s12939-016-0450-5 27716235 PMC5045584

[39] Cabrera-Barona P, Blaschke T, Kienberger S. Explaining accessibility and satisfaction related to healthcare: a mixed-methods approach. Social Indicators Research, 2017; 133(2): 719–739. doi: 10.1007/s11205-016-1371-9 28890596 PMC5569143

[40] Li P, Lu M. Urban Systems: Understanding and Predicting the Spatial Distribution of China’s Population. China & World Economy. 2021, 29(4): 35–62.

[41] Xu F, Wang Z, Chi G, Zhang Z. The impacts of population and agglomeration development on land use intensity: New evidence behind urbanization in China. Land Use Policy. 2020; 95: 104639.

